# Protease Activated Receptor 2 (PAR2) Induces Long-Term Depression in the Hippocampus through Transient Receptor Potential Vanilloid 4 (TRPV4)

**DOI:** 10.3389/fnmol.2017.00042

**Published:** 2017-03-02

**Authors:** Efrat Shavit-Stein, Avital Artan-Furman, Ekaterina Feingold, Marina Ben Shimon, Zeev Itzekson-Hayosh, Joab Chapman, Andreas Vlachos, Nicola Maggio

**Affiliations:** ^1^Department of Neurology, The Chaim Sheba Medical CenterTel HaShomer, Israel; ^2^Department of Neurology and Neurosurgery, Sackler Faculty of Medicine, Tel Aviv UniversityTel Aviv, Israel; ^3^Sagol School of Neuroscience, Tel Aviv UniversityTel Aviv, Israel; ^4^Institute of Anatomy II, Faculty of Medicine, Heinrich-Heine-UniversityDuesseldorf, Germany; ^5^Talpiot Medical Leadership Program, The Chaim Sheba Medical CenterTel HaShomer, Israel

**Keywords:** PAR2, TRPV4, synaptic plasticity, hippocampus, LTD

## Abstract

Protease activated receptors (PARs) are involved in regulating synaptic transmission and plasticity in the brain. While it is well-accepted that PAR1 mediates long-term potentiation (LTP) of excitatory synaptic strength, the role of PAR2 in synaptic plasticity remains not well-understood. In this study, we assessed the role of PAR2-signaling in plasticity at hippocampal Schaffer collateral-CA1 synapses. Using field potential recordings, we report that PAR2-activation leads to long-term depression (LTD) of synaptic transmission through a protein kinase A -dependent, Transient Receptor Potential Vanilloid 4 -mediated mechanism, which requires the activation of *N*-methyl-D-aspartate receptors. These results demonstrate that the effects of PAR2 on synaptic plasticity are distinct from what is observed upon PAR1-activation. Thus, we propose that the activation of different classes of PARs, i.e., PAR1 and PAR2, may set the threshold of synaptic plasticity in the hippocampal network by balancing LTP and LTD.

## Introduction

Synaptic plasticity in the hippocampus underlies the formation of certain forms of memory, including spatial memory (Malenka, 2003; Maggio and Vlachos, 2014). A number of molecules have been postulated to be involved in long-term potentiation (LTP) with recent studies reporting a fundamental role of extracellular proteases in this process (Wlodarczyk et al., 2011; Tsilibary et al., 2014). Among others, protease activated receptors (PAR), which are activated by serine proteases, have been shown to modulate neural excitability and synaptic plasticity (Almonte et al., 2007, 2013; Traynelis and Trejo, 2007; Ben Shimon et al., 2015). PARs belong to a unique family of G protein-coupled receptors (Luo et al., 2007). Their activation is initiated by an irreversible, site-specific proteolytic cleavage in the N-terminal extracellular region, which uncovers a tethered ligand activating Gα_q/11_, Gα_i/o_, or Gα_12/13_ -proteins (Coughlin, 2001; Macfarlane et al., 2001; Traynelis and Trejo, 2007). While PAR1-activation has been shown to modulate synaptic transmission and plasticity through the enhancement of *N*-methyl-D-aspartate receptor (NMDAR) currents (Gingrich et al., 2000; Lee et al., 2007; Maggio et al., 2008; Becker et al., 2014; Vance et al., 2015), and PAR1-deficient animals show alterations in hippocampus-dependent learning and memory processes (Almonte et al., 2007, 2013), the role of PAR2 in neural function and plasticity remains not well-understood.

Unlike other members of the PAR-family, which are effectively activated by thrombin, PAR2 represents a class of trypsin/tryptase-activated receptors (Nieman, 2016). Among other PAR2-activators are tissue kallikreins, coagulation factors VIIa and Xa, and transmembrane serine proteases (Oikonomopoulou et al., 2006; Rezaie, 2014). PAR2 is involved in mediating important biological functions such as inflammation, coagulation, and immunity (Rothmeier and Ruf, 2012; Rezaie, 2014; Bushell et al., 2016). Although PAR2 is detected in the brain (Bushell et al., 2006; Luo et al., 2007; Olianas et al., 2007), and recent work has indicated a role for PAR2 in synaptic plasticity (Lohman et al., 2009; Gan et al., 2011), the molecular signals through which PAR2 affects synaptic transmission and plasticity in the CNS remain unknown.

In the peripheral nervous system PAR2-activation has been linked to neuroinflammation and neuropathic pain (Noorbakhsh et al., 2006; Bao et al., 2014; Tillu et al., 2015; Bushell et al., 2016), through a protein kinase A (PKA)-dependent activation of Transient Receptor Potential Vanilloid 4 (TRPV4) channels (Grant et al., 2007; Chen et al., 2011; Poole et al., 2013). TRPV channels belong to a family of non-selective cation channels that are activated by a wide variety of chemical and physical stimuli (Gunthorpe et al., 2002). While they are highly expressed in sensory neurons in the peripheral nervous system, a number of studies have also reported TRPV expression in the brain (Alter and Gereau, 2008; Shibasaki et al., 2015), though their functions are less well-understood. Recent evidences point toward a role of TRPV channels in hippocampal synaptic plasticity (Gibson et al., 2008; Edwards et al., 2012; Brown et al., 2013). Therefore, we sought to address the hypothesis that PAR2-activation affects synaptic plasticity through TRPV4.

## Materials and Methods

### Chemicals

The following compounds were used at the following concentrations: 10 μM AC55541 (PAR2-agonist, Tocris Bioscience, UK), 10 μM AC264613 (PAR2-agonist, Tocris Bioscience, UK), 50 μM FSLLRY-NH_2_ (PAR2-antagonist, Sigma-Aldrich, Israel), 2 μM RN1747 (TRPV4-agonist, Tocris Bioscience, UK), 10 μM RN1734 (TRPV4-antagonist, Tocris Bioscience, UK), 10 μM RN9893 (TRPV4-antagonist, Tocris Bioscience, UK), 50 μM D(-)-2-amino-5-phosphonovaleric acid (APV, NMDAR-antagonist, Sigma-Aldrich, Israel), 200 μM (±)-a-Methyl-(4-carboxyphenyl)glycine (MCPG, mGluR-antagonist, Sigma-Aldrich, Israel), KT5720 (protein kinase A inhibitor, Tocris Bioscience, UK), GF109203x (protein kinase C inhibitor, Tocris Bioscience, UK). Pharmaceuticals were added to the perfusion medium with special care to prevent changes in temperature, pH, flow rate, or degree of oxygenation of the artificial CSF (aCSF). Handling and disposal of all drugs carried out in accordance to National and Institutional regulations.

### Electrophysiology

This study and protocol was approved by the Sheba Medical Center Institutional Animal Care and Use Committee (1000/15), which adheres to the national law, and NIH rules. Briefly, 4–5 months old male C57BL/6 mice were rapidly decapitated and 350 μm coronal slices containing the dorsal hippocampus were used. Slices were incubated for 1.5 h in a humidified, carbogenated (5% CO_2_ and 95% O_2_) gas atmosphere at 33 ± 1°C and were perfused with a CSF [containing (in mM) 124 NaCl, 2 KCl, 26 NaHCO_3_, 1.24 KH_2_PO_4_, 2.5 CaCl_2_, 2 MgSO_4_, and 10 glucose, pH 7.4] in a standard interface chamber. Recordings were made with a glass pipette containing 0.75 M NaCl (4 MΩ) placed in the stratum radiatum CA1. A cut between CA3 and CA1 was made in order to avoid possible excitability. Stimulation was evoked using a Master 8 pulse stimulator (A.M.P.I., Jerusalem, Israel) and was delivered through two sets of bipolar nichrome electrodes placed on either side of the recording electrode such that two independent stimulation channels were used for each slice. The use of two parallel pathways allowed comparison of the effects of different drug application in the same slice (Maggio and Segal, 2007a,b). Long-term depression (LTD) was induced by low frequency stimulation (LFS) consisting of 1 Hz, 900 pulses, as previously described (Maggio and Segal, 2009). Before applying the protocol, baseline values were recorded at a frequency of 0.033 Hz. Responses were digitized at 5 kHz and stored on a computer. Off-line analysis and data acquisition were performed using Spike 2 software (CED, Cambridge, England). All numerical data are expressed as mean ± SEM, and EPSP slope changes after stimulation were calculated with respect to baseline. There were no systematic differences in the magnitudes of the baseline responses in the different conditions. Unless otherwise indicated, statistical evaluations were performed by applying Student’s *t*-test for paired and unpaired data, as the case may be (Origin 8.0). *p*-values of <0.05 were considered a significant difference between means.

### Immunohistochemistry

The following primary antibodies were used for immunodetection: goat anti-PAR2 (sc-8205, Santa Cruz, 1:25), rabbit anti-TRPV4 (ACC-124, Alomone Labs, 1:50), rabbit anti-PAR2 (APR-032, Alomone Labs 1:500) and mouse anti-GFAP (G3893, Sigma-Aldrich, 1:2000). Hippocampal sections (50 μm) were blocked in 10% normal horse serum in 0.1 M PBS/0.1% Triton for 1 h at room temperature (RT). After 24–48 h incubation at 4°C with the primary antibody (together with 2% normal horse serum), sections were exposed to the appropriate secondary antibody (DyLight^TM^ 488 conjugated affinity purified donkey anti-goat IgG, 1:800; Alexa Fluor 594 AffiniPure donkey anti-rabbit IgG, 1:2000; Alexa Fluor 488 conjugated AffiniPure donkey anti-mouse IgG, 1:400) for 1 h. The sections were then washed, incubated with Hoechst (b1155, Sigma-Aldrich, 1 μg/ml final concentration) for 10 min (to allow nuclear staining), mounted on dry gelatin-coated slides and finally mounted and cover slipped with Flouromount (F4680, Sigma-Aldrich). Slides were imaged with a Leica SP5 confocal microscope and data were acquired and analyzed using a computer assisted image analysis system.

## Results

### PAR2-Activation Induces LTD at Schaffer Collateral-CA1 Synapses

**FIGURE 1:**
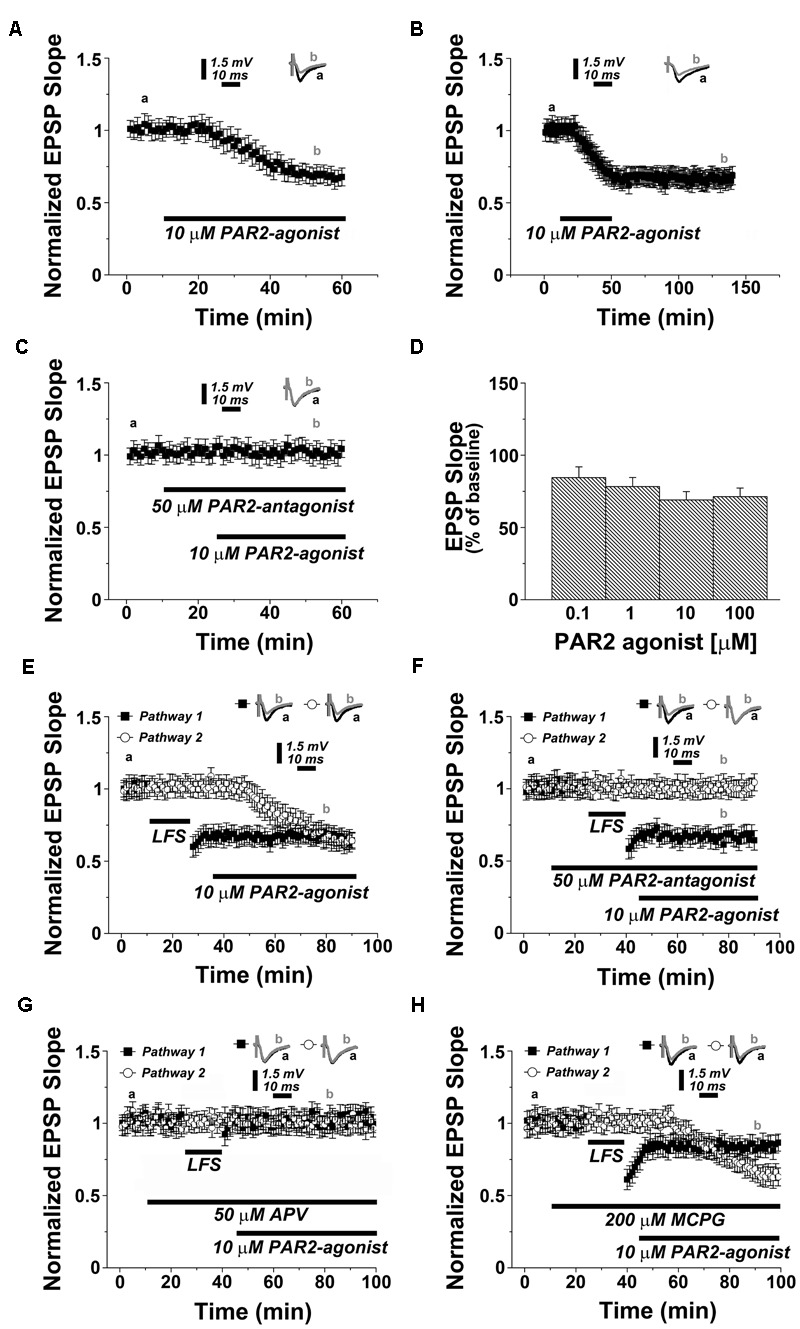
**PAR2-activation induces a depression of synaptic transmission at Schaffer collateral-CA1 synapses in the hippocampus. (A)** Application of PAR2-agonist (10 μM AC55541) causes LTD. **(B)** Removal of the PAR2-agonist (10 μM AC55541) following induction of LTD does not affect the stability of synaptic depression. **(C)** In presence of a PAR2-antagonist (50 μM FSLLRY-NH_2_) the PAR2-agonist (10 μM AC55541) is not able to induce synaptic depression. **(D)** Application of PAR2-agonist (10 μM AC55541) at different concentrations results in similar levels of synaptic depression. **(E)** In a two pathways experimental setting, low frequency stimulation (LFS, 1 Hz, 900 pulses) and PAR2-activation (10 μM AC55541) induce similar levels of LTD. **(F)** LFS-induced LTD is not blocked by the PAR2-antagonist. **(G)** In a two pathways experimental setting, the NMDAR-antagonist (50 μM APV) blocks both LFS-induced LTD and PAR2-induced LTD. **(H)** While the group I mGluR-antagonist MCPG (200 μM) partially affects LFS-LTD it does not influence PAR2-LTD. Averaged EPSP are plotted versus time. Representative traces at indicated times (a, b) are shown on top of each section, *n* = 12 slices for each experiment, refer to text for statistics.

To test for the role of PAR2 in synaptic transmission and plasticity, we first treated acute hippocampal slices with the selective PAR2-agonist AC55541 (10 μM) while recording evoked field potentials of Schaffer collateral-CA1 synapses. A profound depression of synaptic transmission was observed in these experiments reaching 0.73 ± 0.07 of baseline 30 min after bath-application of AC55541 (*p* < 0.001; *n* = 12 slices, **Figure 1A**). Removal of the PAR2-agonist following induction of LTD did not affect the stability of synaptic depression (**Figure 1B**). To confirm the specificity of the PAR2-agonist, we repeated experiments in presence of the selective PAR2-antagonist FSLLRY-NH_2_ (50 μM; washed in 15 min before 10 μM AC55541). Indeed, in this experimental setting PAR2-LTD was not observed (**Figure 1C**). Similarly, activation of PAR2 using a different, specific agonist, i.e., AC264613 (10 μM) also resulted in LTD and this effect was blocked by application of the PAR2-antagonist (**Supplementary Figures S1A,B**, respectively). The effect of the PAR2-agonist (AC55541) was not concentration-dependent, since LTD of similar effects-size was observed when the PAR2-agonist was applied at concentrations of 0.1, 1, and 100 μM (**Figure 1D**). We conclude from these experiments that PAR2-activation induces robust LTD at Schaffer collateral-CA1 synapses.

We then compared the dynamics of PAR2-LTD with LFS-induced LTD. In a two pathway experimental setting, the delivery of a 1 Hz protocol (900 pulses) resulted in a depression of 0.67 ± 0.06 at 30 min, while the PAR2-agonist induced LTD of similar effect-size at the other pathway (0.69 ± 0.07, *p* = 0.378, *n* = 12), without affecting the established LFS-LTD (**Figure 1E**). Moreover, the PAR2-antagonist did not affect the induction and maintenance of LFS-LTD, while preventing PAR2-LTD at the other pathway (**Figure 1F**). However, both forms of LTD required the activation of NMDAR, since 50 μM of the NMDAR-antagonist APV blocked LFS-LTD and PAR2-LTD (**Figure 1G**).

Finally, we tested whether PAR2-mediated LTD is mGluR-dependent by carrying out experiments in presence of the selective mGluR-inhibitor MCPG (200 μM). Consistent with the literature (Maggio and Segal, 2007b; Fitzjohn et al., 2016), LFS-LTD was partially impaired in these experiments (0.82 ± 0.06, *p* < 0.01, *n* = 12, **Figure 1H**). Yet, the induction of PAR2-LTD was not affected by MCPG. Based on these results we conclude that PAR2-LTD requires the activation of NMDAR but not mGluR.

### TRPV4-Activation Induces LTD at Schaffer Collateral-CA1 Synapses

PAR2 is known to mediate its effects, i.e., neuroinflammation and pain in the peripheral nervous system, through the activation of TRPV4 channels (Grant et al., 2007; Chen et al., 2011; Poole et al., 2013). We therefore hypothesized that PAR2 may act on synaptic transmission via TRPV4.

**FIGURE 2:**
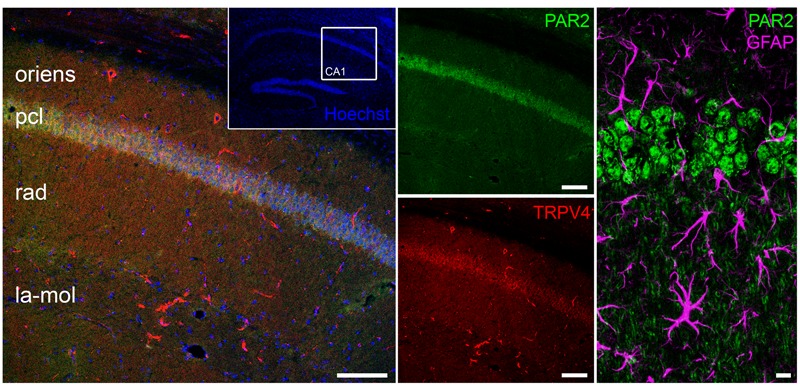
**PAR2 and TRPV4 expression in the hippocampus.** Immunohistochemistry discloses the expression of PAR2 and TRPV4 in the hippocampus. A comparable expression pattern is observed: high levels of PAR2 and TRPV4 are detected in CA1 stratum pyramidale (pcl, pyramidal cell layer; oriens, stratum oriens; rad, stratum radiatum; la-mol, stratum lacunosum-moleculare). No pronounced colocalization between PAR2 and GFAP was detected. Scale bars: 100 and 10 μm, *n* = 9 slices out of three animals.

To test this hypothesis, we first examined PAR2 and TRPV4 expression in the hippocampus. Anatomically matched frontal slices containing the dorsal hippocampus were immunostained for PAR2 and TRPV4. Indeed, both PAR2 and TRPV4 were expressed in the hippocampus. A comparable expression pattern was observed: high levels of PAR2 and TRPV4 were detected in CA1 stratum pyramidale. We did not find a prominent colocalization of PAR2 and the astrocytic marker GFAP in these experiments (**Figure 2**).

**FIGURE 3:**
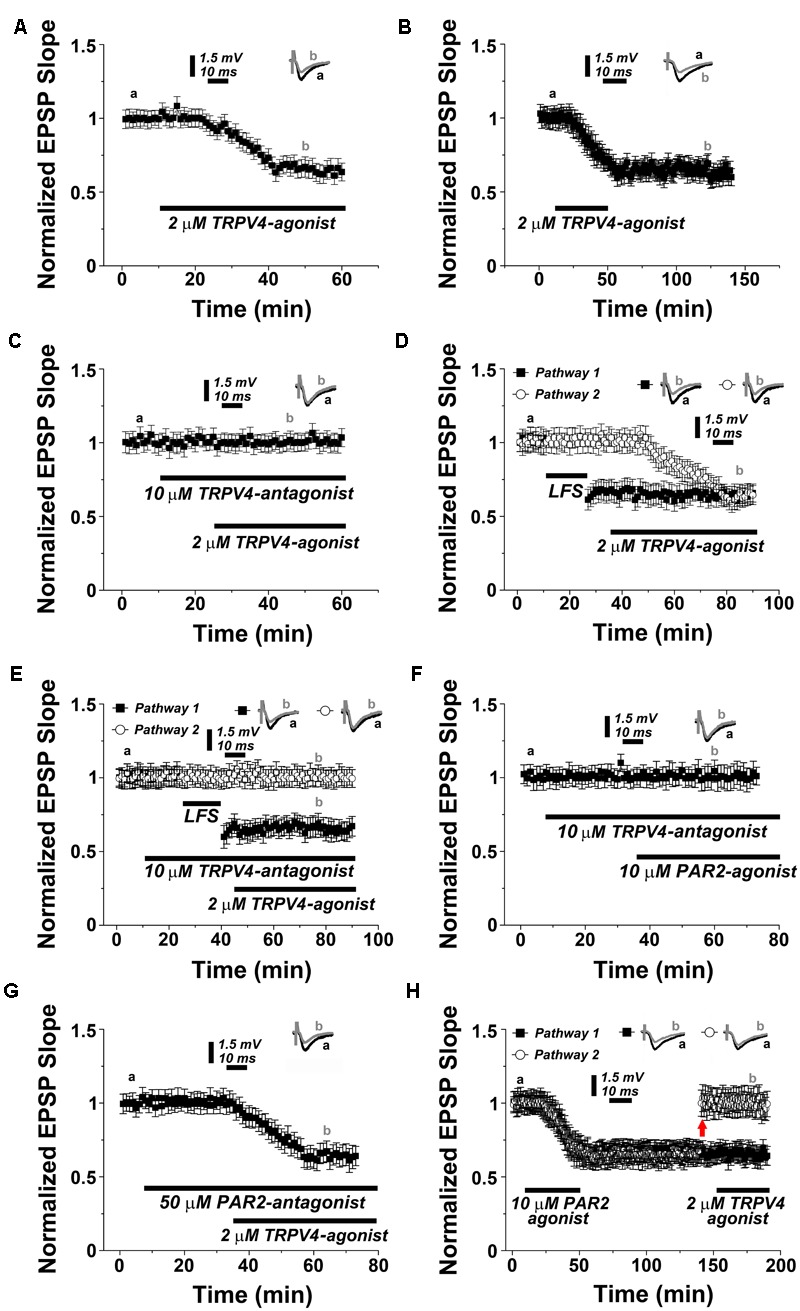
**PAR2 induces LTD through the activation of TRPV4. (A)** Application of TRPV4-agonist (2 μM RN1747) causes LTD. **(B)** Removal of the TRPV4-agonist (2 μM RN1747) following induction of LTD does not affect the stability of synaptic depression. **(C)** In presence of the TRPV4-antagonist (10 μM RN1734) the TRPV4-agonist is not able to induce synaptic depression. **(D)** In a two pathways experimental setting, low frequency stimulation (LFS, 1 Hz, 900 pulses) and TRPV4-agonist application induce similar levels of LTD. **(E)** LFS-induced LTD is not blocked by the TRPV4-antagonist. **(F)** Application of PAR2-agonist (10 μM AC55541) in presence of a TRPV4-antagonist (10 μM RN1734) blocks PAR2-induced LTD. **(G)** Application of TRPV4-agonist (2 μM RN1747) in presence of PAR2-antagonist (50 μM FSLLRY-NH_2_) does not affect TRPV4-induced LTD. **(H)** Once PAR2-agonist mediated LTD is established, the TRPV4-agonist (2 μM RN1747) does not further de-potentiate a second pathway at adjusted response level (upward arrow). Averaged EPSP are plotted versus time. Representative traces at indicated times (a, b) are shown on top of each section, *n* = 12 slices for each experiments, refer to text for statistics.

We then speculated that TRPV4-activation should also result in LTD, similar to what is observed upon PAR2-activation (c.f. **Figure 1**). This prediction was tested by exposing acute hippocampal slices to the TRPV4-agonist RN1747 (2 μM). Indeed, a depression in synaptic transmission occurred, reaching 0.63 ± 0.06% of baseline within 30 min (*n* = 12; **Figure 3A**; c.f. **Figure 1A**). This effect was long lasting as it persisted upon the removal of the TRPV4-agonist (**Figure 3B**). TRPV4-LTD was blocked in the presence of the TRPV4-antagonists RN1734 (10 μM; **Figure 3C**) or RN9893, respectively (**Supplementary Figure S1C**). In two pathway experiments TRPV4-LTD reached similar levels of depression as compared to LFS-LTD (0.66 ± 0.07 versus 0.72 ± 0.05 respectively, *n* = 12, *p* = 0.19, **Figure 3D**), while LFS-LTD was not affected by the TRPV4-antagonist (**Figure 3E**). These experiments disclosed that TRPV4-activation induces robust LTD, similar to what is observed upon PAR2-activation.

### PAR2 Induces LTD through the Activation of TRPV4

In order to test for the interrelation between PAR2- and TRPV4-mediated LTD the following series of experiments was carried out. First, we exposed hippocampal slices to the TRPV4-antagonist before washing in the PAR2-activator (**Figure 3F**). Indeed, PAR2-activation with AC55541 (10 μM) was not able to induce LTD in presence of the TRPV4-inhibitor RN1734 (10 μM). Conversely, treatment with the TRPV4-agonist in presence of the PAR2-antagonist reliably induced LTD (0.64 ± 0.07% of baseline after 30 min, *n* = 12; **Figure 3G**).

We then examined whether sequential PAR2- and TRPV4-activation occlude each other (**Figure 3H**). Upon induction of PAR2-LTD, the PAR2-agonist was removed before washing in the TRPV4-agonist. In these experiments one of the two pathways was manually reported to the baseline value before TRPV4-activation. Indeed, the TRPV4-agonist failed to induce LTD in this setting, suggesting that the pathway was already saturated, i.e., occluded by the prior application of the PAR2-agonist (**Figure 3H**).

**FIGURE 4:**
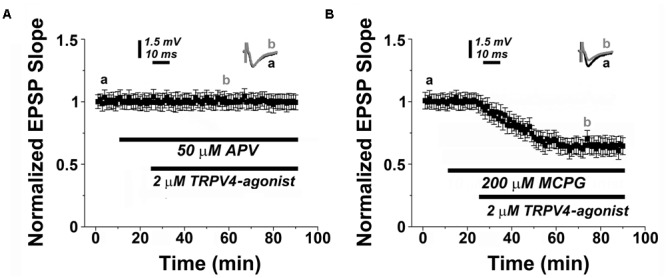
**TRPV4-mediated LTD depends on NMDAR-activity. (A)** Similar to PAR2-induced LTD (c.f., **Figures 1G,H**), the NMDAR-antagonist (50 μM APV) blocks TRPV4 (2 μM RN1747)-induced LTD, while **(B)** application of a TRPV4-agonist (2 μM RN1747) induces LTD in presence of the mGluR-antagonist (200 μM MCGP). Averaged EPSP are plotted versus time. Representative traces at indicated times (a, b) are shown on top of each section.

Finally, we tested whether PAR2- and TRPV4-activation share the same molecular cascade requiring NMDAR but not mGluR activity (c.f. **Figures 1G,H**). Indeed, we observed that TRPV4-activation did not induce LTD in presence of 50 μM APV (*n* = 12, **Figure 4A**), and similar to PAR2-LTD the mGluR-antagonist MCPG (200 μM) was not effective in blocking TRPV4-LTD (0.65 ± 0.04, *n* = 12, **Figure 4B**).

To verify that pharmacological activation of PAR2 or TRPV4 induces a genuine LTD at Shaffer collateral-CA1 synapses, in a separate set of experiments we systematically assessed input/output curves and paired-pulse ratios and we did not find any significant effects on these parameters (**Supplementary Figure S2**). Taken together, we conclude that PAR2 mediates NMDAR-dependent LTD through the activation of TRPV4.

### PAR2-Induced LTD Requires Protein Kinase A

**FIGURE 5:**
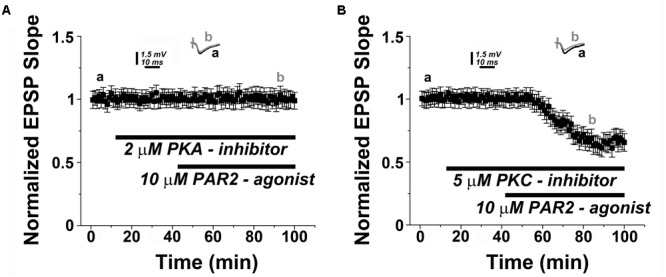
**PAR2-mediated LTD is protein kinase A (PKA)-dependent. (A)** PAR2-agonist (10 μM AC55541) in presence of a PKA-inhibitor (2 μM KT5720) KT5720 (2 μM) fails to induce LTD. **(B)** Application of a protein kinase C (PKC) inhibitor (2 μM GF109203x) does not affect the induction of PAR2-mediated LTD. Averaged EPSP are plotted versus time. Representative traces at indicated times (a, b) are shown on top of each section.

Previous work has indicated that PAR2 acts through PKA to activate TRPV4 in inflammation and pain (Zhao et al., 2015). Hence, to provide further evidence for our major conclusion, we decided to test whether PKA is involved in mediating PAR2-LTD. This hypothesis is relevant also for the known role of PKA as a canonical signaling molecule associated with hippocampal LTD (Collingridge et al., 2010; Hell, 2016; Sanderson et al., 2016). Indeed, in presence of the PKA-inhibitor KT5720 (2 μM) PAR2-activation failed to induce LTD (**Figure 5A**). This effect was specific, since pharmacological inhibition of protein kinase C (PKC), an additional molecule reported to be involved in LTD (Collingridge et al., 2010), had no apparent effect on PAR2-LTD (**Figure 5B**).

## Discussion

The present study addresses the role of PAR2-mediated signaling pathways in synaptic plasticity of central neurons. Based on our results we propose a model in which PAR2-activation induces (PKA-dependent) TRPV4-mediated LTD, which requires NMDAR-activity. Hence, our study is the first to report that PAR2 induces LTD at central synapses via TRPV4.

Work from recent years has identified an important role of PARs in the regulation of synaptic plasticity in the central nervous system. In this context the role of PAR1 has been extensively studied (Ben Shimon et al., 2015). It has been demonstrated that PAR1-activation induces LTP, which affects the ability of neurons to express further plasticity (Maggio et al., 2008; Stein et al., 2015). The results of the present study now demonstrate that in stark contrast to PAR1, PAR2 induces LTD (see also Gan et al., 2011). Considering that different proteases activate PAR1 and PAR2, it is interesting to hypothesize that PAR1- and PAR2-mediated synaptic plasticity could be the target of distinct protease signaling pathways, which aim at setting and modulating the threshold and direction of synaptic plasticity by balancing LTP and LTD.

This suggestion is of considerable relevance in the context of neurological diseases associated with the break-down of the blood brain barrier and/or increased brain proteases levels, e.g., brain thrombin concentrations (Chapman, 2006; Maggio et al., 2013a; Itzekson et al., 2014; Bushi et al., 2015; Deselms et al., 2016; Itsekson-Hayosh et al., 2016). Evidence has been provided that thrombin-induced PAR1-mediated LTP saturates and thus occludes the ability of neurons to express synaptic plasticity. Accordingly, it has been hypothesized that PAR1-inhibitors may improve the ability of neurons to express plasticity by preventing the pathological induction of LTP (Maggio et al., 2013b, 2014; Becker et al., 2014). However, it has been also recognized that the effects of PAR1 on synaptic plasticity can differ depending on the activating proteases and the concentration of activating ligands – a phenomenon termed ‘biased agonism’ (Grimsey et al., 2011). For example low concentrations of thrombin, or a specific PAR1-agonist, improve the ability of neurons to express synaptic plasticity without inducing LTP *per se* (Maggio et al., 2013b, 2014). Thus, while in a recent study we were not able to detect adverse effects of prolonged PAR1-inhibition on dendritic plasticity of neurons (Schuldt et al., 2016), it is conceivable that pharmacological inhibition of PAR1 may not only exert positive effects on neural plasticity.

A similar dose-dependent effect of PAR2-activation on synaptic plasticity was not detected in the present study, as distinct concentrations of the specific PAR2-agonist reliably induced LTD. Although these results do not rule out an impact of ‘biased agonism’ on PAR2-mediated synaptic plasticity, it is interesting to speculate that PAR2-induced synaptic depression may counteract detrimental effects of PAR1-LTP in a ‘dose-independent manner.’ Such interaction between PAR1 and PAR2 may follow the rules of metaplasticity (Abraham, 2008; Hulme et al., 2013) and thus PAR2-LTD could robustly reverse or re-set the threshold of synaptic plasticity under conditions of a thrombin/PAR1-LTP mediated saturation of synaptic plasticity. Whether PAR2-agonists could prove suitable for the treatment of stroke and other diseases associated with increased brain thrombin levels or PAR1-activity is matter of current investigations. Apparently, in this context the role of distinct proteases and PARs in mediating metaplasticity, i.e., balancing neural plasticity under physiological and pathological conditions, needs to be determined.

Regardless of these considerations our work demonstrates that PAR2-LTD is mediated via TRPV4. Previous work has linked TRPV channels to the regulation of synaptic plasticity (Kauer and Gibson, 2009). It has been shown for example that TRPV1 mediates LTD of excitatory synapses on interneurons through an endocannabinoid-mGluR-dependent mechanism (Brown et al., 2013). Whether this mechanism is also mediated by PAR2 is not known. Notably, our data show that PAR2-TRPV4-mediated LTD of excitatory synapses on hippocampal principal neurons is not mGluR-dependent. These findings raise important questions regarding the cellular and subcellular distribution of PAR2 and distinct TRPVs, and how this distribution may affect the specific response to the exposure of distinct proteases. In this context the slow kinetics of PAR2/TRPV4-mediated LTD, compared to the one observed in the LFS induced LTD, may possibly depend on a delay by which PAR2-activation promotes glutamate release from the synaptic terminals via TRPV4 (Kauer and Gibson, 2009; Hunt et al., 2012). Astrocytic PAR2 expression (Bushell et al., 2006) could play an important role in orchestrating this process. However, in the present study we were not able to detect a robust astrocytic PAR2-signal in the hippocampus. Thus additional work is required to determine the precise distribution of PAR2 and TRPV in distinct brain regions, cell types and neural compartments in order to learn more about how PAR2-signaling pathways regulate the ability of neurons to express synaptic plasticity through TRPV-activation.

While our results stand along with the work from TRPV4-deficient mice, which show impaired neuronal excitability and altered social as well as depressed behavior (Shibasaki et al., 2015), the precise mechanisms how NMDARs mediate PAR2-TRPV4-LTD and the functional relevance of this mechanisms for physiological and pathological brain functions is unclear. At this point we can only state that PAR2-TRPV4-mediated LTD is NMDAR- and not mGluR-dependent. Considering that a link between PAR2 and TRPV4 has already been established in the peripheral nerve system (Grant et al., 2007; Chen et al., 2011; Poole et al., 2013), where proteases that activate PAR2 sensitize TRPV-channels, which amplifies the proinflammatory and hyperalgesic actions of proteases (Noorbakhsh et al., 2006; Poole et al., 2013), our study provides first evidence that similar interactions, i.e., (PKA-dependent) PAR2-mediated TRPV4 sensitization is also relevant in mediating and modulating synaptic plasticity in the central nervous system.

## Author Contributions

Conceived the study: NM, AV, JC. Conducted experiments and analyzed data: ES-S, AA-F, EF, MBS, ZI-H, NM. Wrote the paper: NM, AV.

## Conflict of Interest Statement

The authors declare that the research was conducted in the absence of any commercial or financial relationships that could be construed as a potential conflict of interest.

## Supplementary Material

FIGURE S1 **Specificity of PAR2- and TRPV4-mediated LTP. (A)** Application of a different PAR2-agonist (10 μM AC264613) resulted in similar levels of LTD. **(B)** In presence of a PAR2-antagonist (50 μM FSLLRY-NH_2_) the PAR2-agonist (10 μM AC264613) is also not able to induce synaptic depression; **(C)** A different TRPV4-antagonist (10 μM RN9893) also blocked TRPV4-agonist (2 μM RN1747) induced LTD. Averaged EPSP are plotted versus time. Representative traces at indicated times (a, b) are shown on top of each section.

FIGURE S2 **Effects of PAR2- and TRPV4-agonists on Input/Output curves and paired pulse responses.** Neither PAR2-agonist (10 μM AC55541) nor TRPV4-agonist (2 μM RN1747) affected presynaptic volley **(A)** and EPSP responses **(B)** when Input/Output curves were tested. **(C)** Paired pulses responses were not affected by PAR2-agonist (10 μM AC55541) or TRPV4-agonist (2 μM RN1747).
