# Supplementary figures and images for: Protease Activated Receptor 2 (PAR2) Induces Long-Term Depression in the Hippocampus through Transient Receptor Potential Vanilloid 4 (TRPV4)
Source: Front Mol Neurosci. 2017 Mar 2;10:42. doi: 10.3389/fnmol.2017.00042 (PMC5332813; doi:10.3389/fnmol.2017.00042)

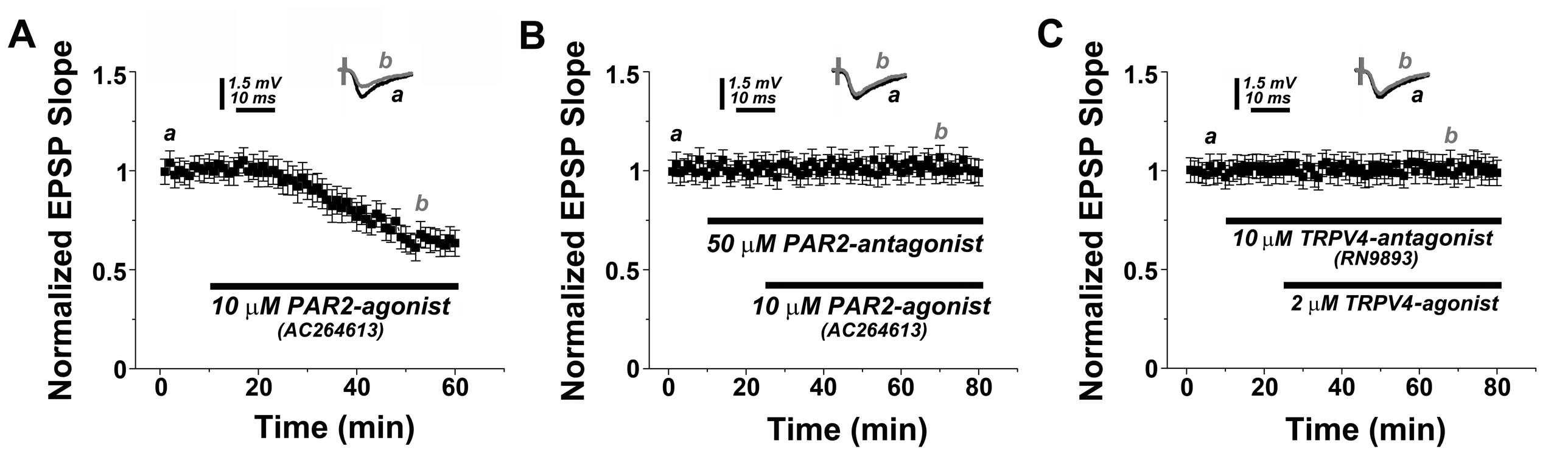

Supplement: FIGURE S1 — Specificity of PAR2- and TRPV4-mediated LTP. (A) Application of a different PAR2-agonist (10 μM AC264613) resulted in similar levels of LTD. (B) In presence of a PAR2-antagonist (50 μM FSLLRY-NH2) the PAR2-agonist (10 μM AC264613) is also not able to induce synaptic depression; (C) A different TRPV4-antagonist (10 μM RN9893) also blocked TRPV4-agonist (2 μM RN1747) induced LTD. Averaged EPSP are plotted versus time. Representative traces at indicated times (a, b) are shown on top of each section. [file Image_1.TIF]

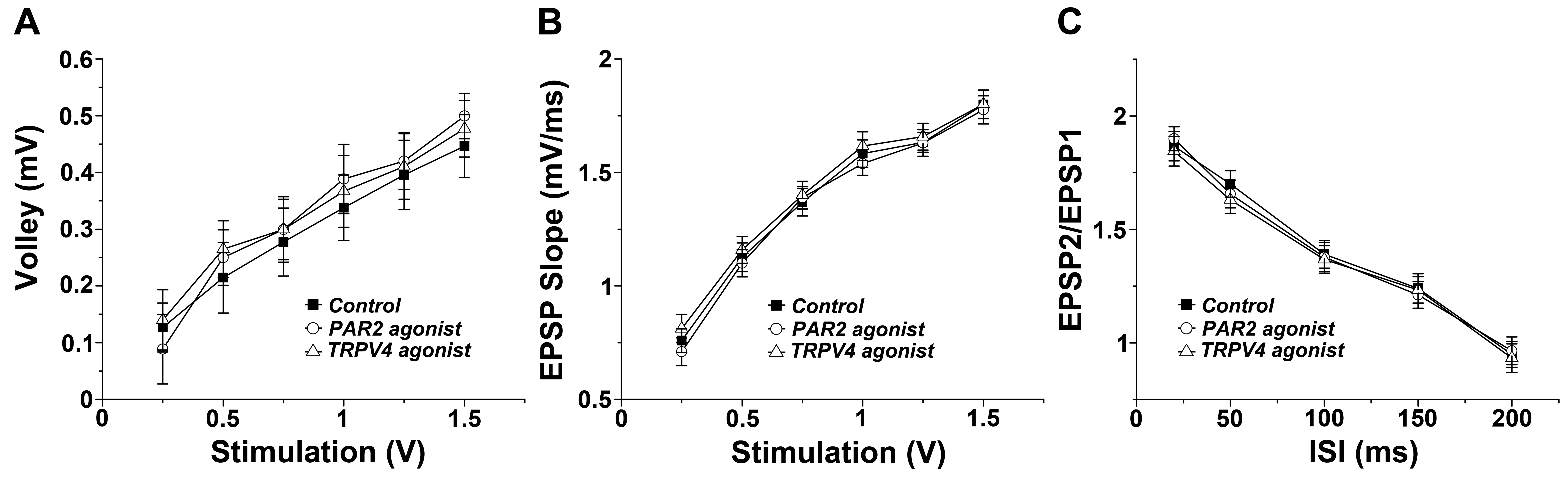

Supplement: FIGURE S2 — Effects of PAR2- and TRPV4-agonists on Input/Output curves and paired pulse responses. Neither PAR2-agonist (10 μM AC55541) nor TRPV4-agonist (2 μM RN1747) affected presynaptic volley (A) and EPSP responses (B) when Input/Output curves were tested. (C) Paired pulses responses were not affected by PAR2-agonist (10 μM AC55541) or TRPV4-agonist (2 μM RN1747). [file Image_2.TIF]
